# Treatment of Early-Stage Pressure Ulcers by Using Autologous Adipose Tissue Grafts

**DOI:** 10.1155/2014/817283

**Published:** 2014-04-10

**Authors:** Giovanni Francesco Marangi, Tiziano Pallara, Barbara Cagli, Emiliano Schena, Francesco Giurazza, Elio Faiella, Bruno Beomonte Zobel, Paolo Persichetti

**Affiliations:** ^1^Department of Plastic and Reconstructive Surgery, “Campus Bio-Medico di Roma” University, Via A. del Portillo 200, 00128 Rome, Italy; ^2^Laboratory of Research in Biomedical Instrumentation and Measurements, “Campus Bio-Medico di Roma” University, Via A. del Portillo 200, 00128 Rome, Italy; ^3^Division of Radiology, “Campus Bio-Medico di Roma” University, Via A. del Portillo 200, 00128 Rome, Italy

## Abstract

Assessing pressure ulcers (PUs) in early stages allows patients to receive safer treatment. Up to now, in addition to clinical evaluation, ultrasonography seems to be the most suitable technique to achieve this goal. Several treatments are applied to prevent ulcer progression but none of them is totally effective. Furthermore, the in-depth knowledge of fat regenerative properties has led to a wide use of it. With this study the authors aim at introducing a new approach to cure and prevent the worsening of early-stage PUs by using fat grafts. The authors selected 42 patients who showed clinical and ultrasonographic evidence of early-stage PUs. Values of skin thickness, fascial integrity, and subcutaneous vascularity were recorded both on the PU area and the healthy trochanteric one, used as control region. Fat grafting was performed on all patients. At three months, abnormal ultrasonographic findings, such as reduction of cutaneous and subcutaneous thickness, discontinuous fascia, and decrease in subcutaneous vascularity, all were modified with respect to almost all the corresponding parameters of the control region. Results highlight that the use of fat grafts proved to be an effective treatment for early-stage PUs, especially in the care of neurological and chronic bedridden patients.

## 1. Introduction

Pressure ulcers (PUs) are necrotic lesions affecting the epidermis, the dermis, and the subcutaneous layers, extending also to the underlying muscles and bones. They are mainly due to the ischemia of both superficial and deep tissues, which is caused by high and/or prolonged capillary compression [1–5]. Studies performed to date have confirmed that the sacral area is the most frequently involved one [6]. PUs prevalence in Europe (stages I–IV) is estimated at 18.1% or 10.5%, when stage I ulcers are excluded [7, 8]; the highest incidence is found to be among the elderly and bedridden patients, especially those who are hospitalized or in long-term care settings [9, 10]. The most widely used staging system was proposed by the National Pressure Ulcer Advisory Panel (NPUAP) in 1989 and then updated in 2007 [11]. Nowadays, a specific landmark for the treatment of PUs does not exist yet. The approaches vary according to the stage of the lesion and any complications that may result. Recent experimental studies have shown that ultrasound (US) examination of subcutaneous deep tissues in high-risk areas is an effective method for early detection of PUs, and it allows the prediction of their deepening towards the underlying tissues. Aoi et al. [12] and Yabunaka et al. [13] reported specific ultrasonographic abnormal findings localized in the subcutaneous fat tissue, both in deep tissue injury and in stage I PU. These signs are reversible with wound healing, unlike tissue necrosis which shows characteristics of irreversibility, as seen in advanced PUs stages. Therefore, the identification of PUs in their early stages would allow physicians to establish an adequate treatment plan at the very beginning of these lesions. Recently, fat grafting has already been performed to treat bedsores; however, its use is restricted to advanced stages [14]. The idea for this study derives from the accredited and established belief that adipose tissue is a great source of adult-derived stem cells (ASCs), capable of differentiating into several cellular lines [15] and regenerating damaged tissues. In this study, the authors aimed at evaluating the potential benefits of autologous adipose tissue grafts in the treatment of early-stage PUs (DTI and stage I). Changes in superficial and deep tissues after fat grafting were also evaluated, in terms of superficial and subcutaneous thickness, tissue vascularization, and integrity of fascia superficialis.

## 2. Materials and Methods

From January 2011 to December 2012 we performed a longitudinal prospective experimental study at the “Campus Bio-Medico di Roma” University, Italy. In the department of plastic and reconstructive surgery, patients with visible stage I PUs, signs of tissue damage, or at high-risk of developing a bedsore underwent wound assessment. High-resolution US examination was performed by the department of radiology of the same hospital. All patients underwent a preliminary screening, in order to check for strict adherence to inclusion and exclusion criteria. Patients were included according to the following parameters: age between 18 and 65 y and presence of at least one PU at stage I or a deep tissue injury (according to the updated NPUAP staging system), localized on the sacral and/or ischial areas and resistant to common ulcer management (repositioning, nutritional support, appropriate support surfaces, cleansing solutions, and advanced wound care dressings). Patients with a bedsore stage higher than one, with contraindications to surgical procedure, or those who did not sign the informed consent for the study were all excluded. Photographs were taken for evaluating the investigated areas during the follow-up period. The study protocol conformed to the ethical guidelines of the 1975 Declaration of Helsinki was approved by the ethics committee, and the results from this assessment have been documented and stored on authors' database.

### 2.1. Ultrasonography

All patients who satisfied all inclusion criteria underwent US examination performed by the same radiologist, familiarly experienced on the use of this technique, for the assessment of skin and subcutaneous tissues. Subjects were investigated in the prone position for PUs analysis and in the lateral one for unaffected great trochanter (homolateral side) scan; the latter was used as a control region. In authors' opinion, in fact, the proximity of the trochanteric zone to the investigated areas and the good ultrasound visualization of its subcutaneous tissue make this area the most suitable element of comparison. Each US examination lasted approximately 15 minutes. Linear-array 7.5–15/13.5 MHz probes (Philips HD11 XE, preset soft tissues) were used. The following parameters have been investigated by US assessment on both sides: skin and subcutaneous thickness, continuity or discontinuity of fascia superficialis, and vascularization ratio between subcutaneous layer and the underlying muscular one. The latter was evaluated using the Doppler technique and it was expressed according to a scale of 5 grades (Table 1); 0 was considered a normal value.

### 2.2. Fat Grafting

To ensure consistency, uniformity, quality of results and procedures, and complying with the Good Clinical Practice, the surgical procedure was performed by the same team and with the same technique. After disinfecting with iodopovidone, Klein's modified anesthetic solution was first infiltrated in the donor site (about 1 cc per cc of expected lipoaspirate). To harvest adipose tissue, liposuction from common suitable donor sites (abdomen, hips, or crural region) was performed using a two-holed blunt cannula connected to a 10 mL Luer-Lok syringe. The syringes were then placed into a sterile centrifuge and spun at 3000 rpm for 1 minute. After removing the syringes from the centrifuge, the liquid and oily components were drained leaving only the solid layer, which accounts for the regenerative elements. PU lipofilling was then performed: 1 cc of fat per cm^2^ of the recipient area was injected in the deep subcutaneous layer, where ischemic injury originates, using a 3 mm lipostructure cannula connected to the 10 mL Luer-Lok syringe.

The ultrasonographic criteria investigated during the screening visit were reassessed after 3 months. Collecting data was performed with an ad hoc case report form. Data were expressed as mean value ± SD or percentage, where appropriate. Results were analyzed using the paired* t*-test for parametric values and Fisher's exact test for nonparametric ones. Significance was determined by a* P* value < 0.05.

## 3. Results and Discussion

After screening, 42 patients satisfied inclusion criteria, as described in Table 2. After a three-month follow-up, results for the different parameters of investigation are given below.

### 3.1. Skin and Subcutaneous Thickness

**Figure 1:**
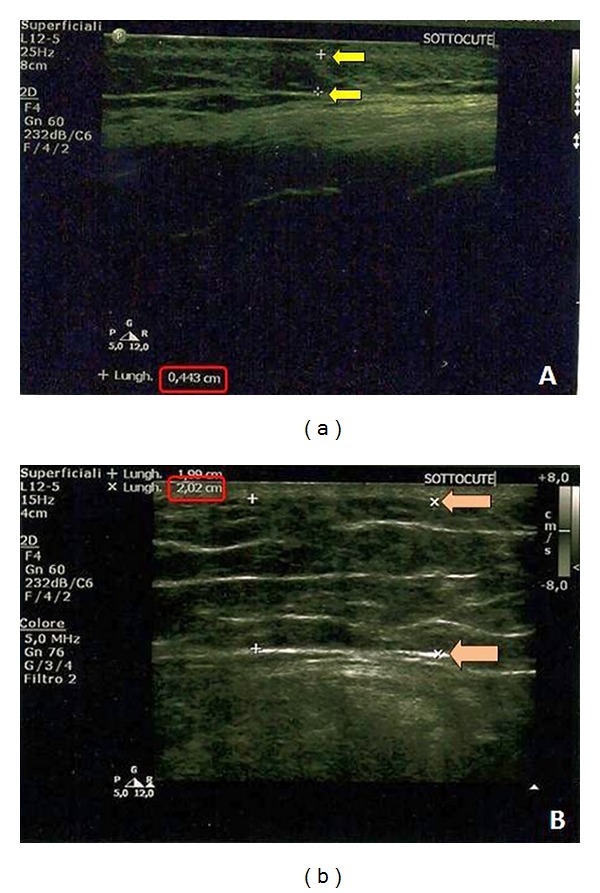
(a) Preoperative subcutaneous tissue ultrasonography; (b) 3-month postoperative subcutaneous tissue ultrasonography. Shown by the arrows is the increase of the subcutaneous thickness.

**Figure 2:**
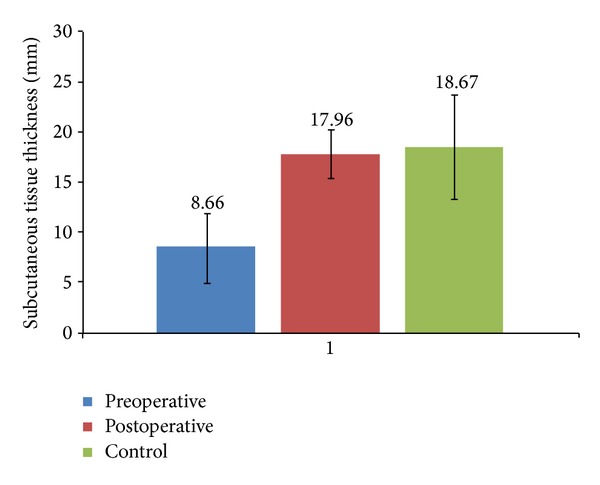
Mean values of subcutaneous thickness.

Following the surgical treatment, the PUs skin thickness values (mean 1.40 ± 0.32 mm), which were lower at baseline than those of the control area in all cases, increased even though not significantly (*P* = 0.07). Ultrasound examination of the subcutaneous tissue, on the other hand, showed a statistically significant increased thickness if compared to preoperative analysis (mean value 8.66 ± 3.42 mm), reaching values close to the control area (mean value 18.67 ± 5.16 mm,* P* < 0.001) (Figures 1(a), 1(b), and 2). No increase in thickness was observed only in one case.

### 3.2. Vascularization Ratio

**Figure 3:**
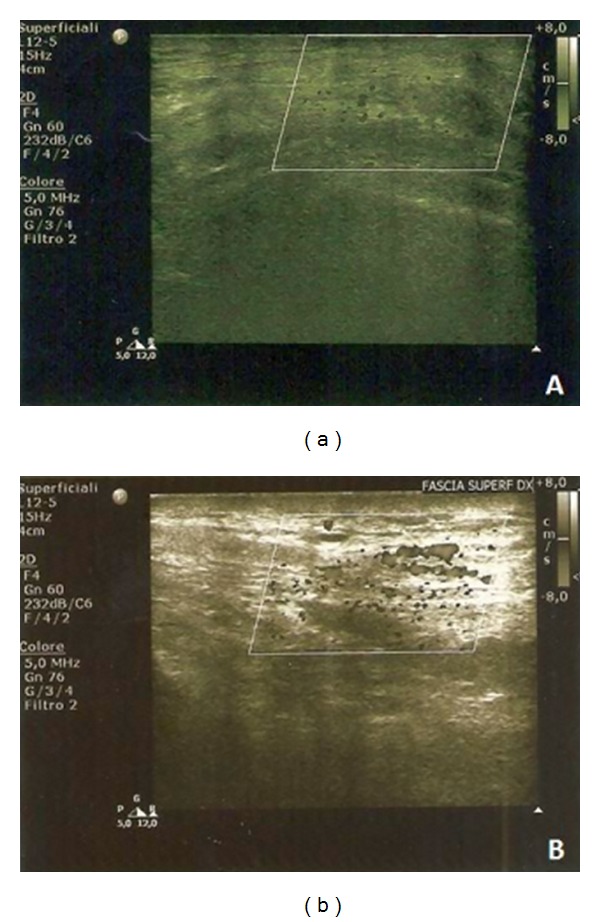
(a) Preoperative Doppler ultrasonography (V0); (b) 3-month postoperative Doppler ultrasonography (V1).

The evaluation of the vascularization ratio, between the subcutaneous and the underlying muscular layers, confirmed the neovascularization properties of the adipose tissue. At baseline, the subcutaneous tissue presented a decreased vascularization compared to the underlying muscular tissue. After fat grafting, subsequent US and Doppler examination showed an increased vascular spots density (Figures 3(a) and 3(b)) almost in all cases (*P* = 0.07).

### 3.3. Fascia Superficialis

**Figure 4:**
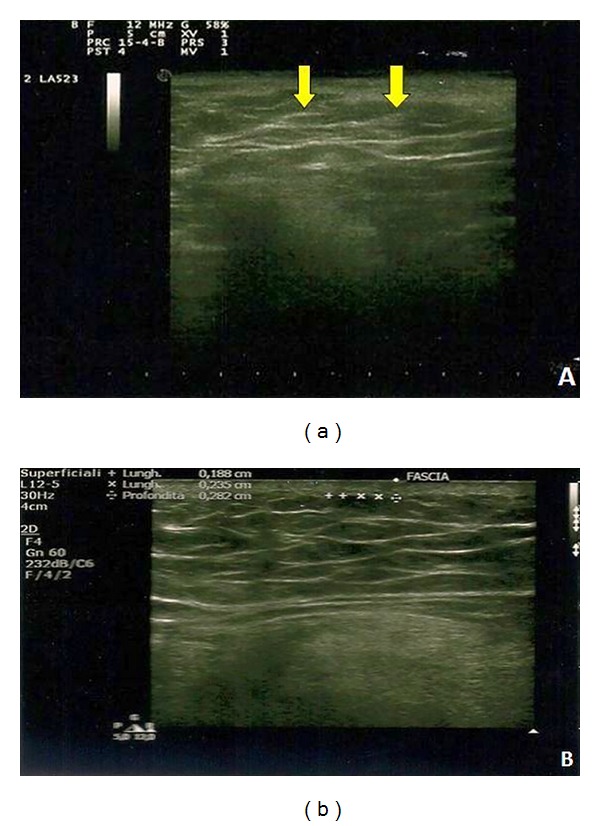
(a) Preoperative ultrasonography showed discontinuous fascia; (b) 3-month postoperative ultrasonography showed continuous fascia.

At screening, discontinuous fascia was found in 79% of patients (Figure 4(a)). This US finding was probably due to the presence of subcutaneous fat edema which prevented its correct visualization. After fat grafting, as the wound healed and inflammation rate decreased, ultrasound images showed an intact continuous fascia superficialis in 85% of patients (*P* < 0.001) (Figure 4(b)).

**Figure 5:**
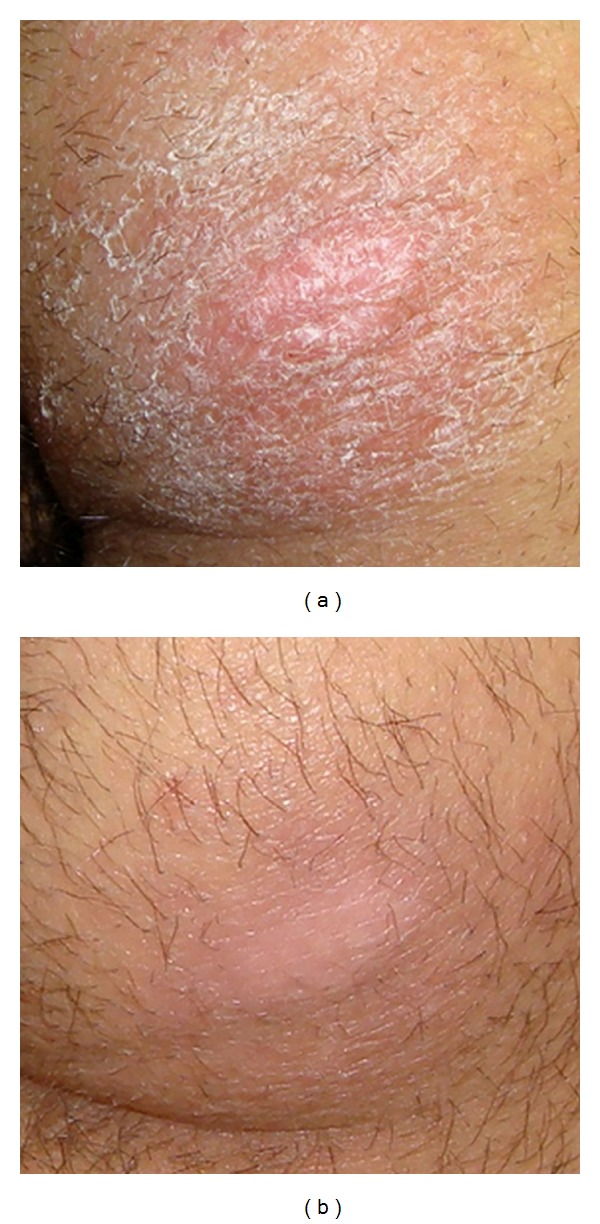
(a) Clinical preoperative appearance; (b) clinical postoperative appearance.

Furthermore, the skin over the grafted area showed a decrease (*n* = 18) or disappearance (*n* = 10) of fixed erythema, checked by daily photographs and glass plate compression, in the whole stage I PU group [16] and increased elasticity, distensibility, and softness at palpation in all cases (Figures 5(a) and 5(b)). No significant complications were reported.

PUs are a serious medical condition that requires challenging care, especially with regard to the patient management [17]. Moreover, PUs are a cause of discomfort for patients, especially when they occur in an advanced stage [18]. Nowadays, a specific landmark for the treatment of bedsores does not exist yet. Common medical approaches are [19–22] advanced wound care dressings, infusion feeding therapies, antibiotics, and accurate local cleansing (e.g., Vacuum Assisted Closure) [23]; on the other hand, surgical reconstructive techniques, ranging from skin grafts to composite, pedicled, and free flaps, are often the treatment of choice when spontaneous recovery is not possible, especially when wound healing is slow and because superinfections are likely to develop [24, 25]. However, surgical approach is invasive and expensive, requiring long periods for rehabilitation, leading to further deterioration of patients' quality of life, and is not recurrence-free. Therefore, the possibility of treating the earliest stages of PUs and thus preventing their worsening is the most desirable solution to this challenging problem. Recent studies reported the usefulness of US examination in the diagnoses of deep tissue damage and in predicting the deterioration of bedsores [12, 13, 26, 27]; these results lead us to support the hypothesis above. It is now common to consider fat grafting as a minimally invasive surgical procedure; furthermore not only does it allow correcting volume defects, but also it contributes to local tissue regeneration [28]. The latter is highly dependent on survival, proliferation, and differentiation properties of ASCs, which have proved to be pluripotent stem cells, present in the so-called stromal vascular fraction (SVF) of lipoaspirate, and able to differentiate into several cellular lines (adipocytes, chondrocytes, osteocytes, myocytes, hepatocytes, endothelial, and neuronal cells) [29–34]. Experimental studies have also confirmed the proangiogenetic characteristics of ASCs [35–38]. Therefore, in a condition of decreased adipose tissue and altered architecture of subcutaneous tissues, along with local ischemia, all typical characteristics of early-stage PUs, autologous adipose tissue grafting may be, in authors' opinion, a possible treatment approach. At present, in the literature, no indications are recommended for this purpose. Moreover, the regenerative properties of adipose tissue have been confirmed by the increased thickness of skin and subcutaneous tissue, the repair of the fascia superficialis continuity, and the regeneration and improvement in skin texture, elasticity, and trophism, achieved after treatment. Furthermore, the proangiogenetic properties of fat translated in an increased vascularity of subcutaneous tissue, induced by fat grafts rich in ASCs, as shown by Doppler US examination.

## 4. Conclusions

According to the authors' statements, the protocol described may be an effective approach for the treatment of early-stage PUs, thanks to the minimally invasive surgical procedure such as fat grafting (which takes advantage of the regenerative properties of adipose tissue) associated with the ultrasonography (cost-effective, reproducible, and easy-to-perform method). Moreover, results reveal the idea that this method may be a multistep treatment in patients chronically subject to a pressure stimulus, without implying a worsening of underlying medical conditions.

This study showed that autologous adipose tissue grafts are an effective treatment in patients with stage I PU or DTI. Furthermore, results achieved with this approach in treated tissues justify the role of prevention from PUs worsening to more advanced stages. Nevertheless, considering the importance of Evidence Based Medicine and the imperative need for more objective methods, further randomized studies with a longer follow-up period are needed to firmly establish the validity of the adopted method and the possibility of including it among the measures of gold standard treatment for certain early-stage PUs.

## Figures and Tables

**Table 1:** Vascularization ratio between subcutaneous and muscular layers. 0 was considered a normal value.

Grade	Vascularization ratio
+2	Subcutaneous layer >> muscular layer
+1	Subcutaneous layer > muscular layer
0	Subcutaneous layer = muscular layer
−1	Subcutaneous layer < muscular layer
−2	Subcutaneous layer << muscular layer

**Table 2:** Patients characteristics.

Patients	42
Mean age	54 ± 10 y
BMI	26 ± 3
Bedsore localization	
Ischium	18
Sacrum	24
Primary disease	
Paraplegia	13
Spina bifida	3
Multiple Sclerosis	8
Poststroke immobilization	5
Tetraplegia	4
Diabetes	9
PU stage	
DTI	14
Stage I	28
